# Psychometric Properties of the Abbreviated Version of the Dual School Climate and School Identification Measure–Student (SCASIM-St15) among Adolescents in China

**DOI:** 10.3390/ijerph192416535

**Published:** 2022-12-09

**Authors:** Yanqiu Yu, Joyce Hoi-Yuk Ng, Anise M. S. Wu, Juliet Honglei Chen, Deborah Baofeng Wang, Guohua Zhang, Mengni Du, Dajin Du, Mingxuan Du, Joseph T. F. Lau

**Affiliations:** 1 Department of Preventive Medicine and Health Education, School of Public Health, Fudan University, Shanghai 200030, China; 2 Key Laboratory of Public Health Safety, Fudan University, Shanghai 200030, China; 3 Center for Health Behaviours Research, Jockey Club School of Public Health and Primary Care, The Chinese University of Hong Kong, Hong Kong SAR, China; 4 Department of Psychology, Faculty of Social Sciences, University of Macau, Macao, China; 5 Centre for Cognitive and Brain Sciences, Institute of Collaborative Innovation, University of Macau, Macao, China; 6 Department of Psychology, Zhejiang Sci-Tech University, Hangzhou 311100, China; 7 Zhejiang Provincial Clinical Research Center for Mental Disorders, The Affiliated Wenzhou Kangning Hospital, Wenzhou Medical University, Wenzhou 325000, China; 8 School of Mental Health, Wenzhou Medical University, Wenzhou 325000, China; 9 Teaching and Research Center, Bureau of Education, Linhai 317000, China; 10 School of Public Health, Zhejiang University, Hangzhou 311100, China

**Keywords:** school climate, school identification, validation, psychometric properties, China, adolescents

## Abstract

School climate and school identification are two distinct yet closely interrelated components of school environment; both are associated with adolescents’ multiple health behavioral changes. The 15-item Abbreviated version of the Dual School Climate and School Identification Measure–Student (SCASIM-St15) and its 5-factor model simultaneously and separately assess these two constructs. This study validated the Chinese version of SCASIM-St15 among 1108 students from junior middle schools, senior middle schools, and vocational high schools in Taizhou city, Zhejiang, China, via an anonymous, self-administered cross-sectional survey. Confirmatory factor analysis supports the 5-factor model of the original SCASIM-St15 with a satisfactory model fit. Its four factors (i.e., student–student relations, staff–student relations, academic emphasis, and shared values and approach) assess school climate; its fifth factor assesses school identification. The subscales of the SCASIM-St15 demonstrate good psychometric properties, including measurement invariance (across sex and school type), good internal consistency, an absence of floor effect, and good external validity with four external variables (depression, peer victimization, classmate support, and teacher–student relationship). However, some substantial ceiling effects were observed. The five subscales differ significantly across the school types but not between males and females. The validated SCASIM-St15 can be applied to simultaneously understand school climate/school identification among Chinese adolescents, which may greatly facilitate future related observational and intervention research.

## 1. Introduction

Unhealthy lifestyles (e.g., physical inactivity, eating disorders), behavioral problems (e.g., smoking, gaming addiction, bullying), and mental distress (e.g., depression) are prevalent in adolescents and have long-term negative consequences. The socio-ecological model postulates that structural factors (including social environment) are important, apart from factors on interpersonal and individual levels [1]. However, research on the structural factors of health outcomes (e.g., multiple health behaviors) seems to be less emphasized. Behavioral health theories also highlight the important relationship between environment and adolescent behavioral health. For instance, the reciprocal determinism construct of the Social Cognitive Theory explains that the environment interacts with individual characteristics to determine health-related behaviors and health outcomes [2]. To adolescents, the school environment is certainly of the utmost significance, as it is the major setting where education, social interactions, socialization, peer influences, and even health promotion take place.

School climate is an important component of the school environment. It refers to the “pattern of students’ parents’ and school personnel’s experience of school life [that] reflects norms, goals, values, interpersonal relationships, teaching and learning practices, and organizational structures (p. 182)”. Cohen et al. suggest four dimensions, including safety, relationships (with staffs and peers), teaching and learning (e.g., supportive teaching practices), and institutional environment (e.g., school connectedness) [3]. Instead of focusing on physical resources and facilities, school climate emphasizes the psychosocial school atmosphere and intergroup relationships (e.g., student–student relationships and teacher–student relationships) [4]. Extant literature has documented the importance of school climate in affecting adolescent behavioral health and mental health. For instance, a supportive school climate predicts lower levels of school bullying [5,6], stress, and depressive symptoms [7]. A meta-analysis of longitudinal studies also reported that a positive school climate is associated with fewer problematic behaviors in schools (e.g., school delinquency and general conduct problems) [8].

School identification is another key component of the school environment. It refers to the sense of belonging to the school and care for school-related goals [9]. School identification is initially developed via an internalization process; i.e., teachers’ and students’ engagements with the school start with the development of extrinsic motivations (e.g., praise from teachers and fun in playing with peers) and then intrinsic drives to foster belongingness [9]. School identification is associated with academic achievement [4] and in-school and out-of-school delinquent behaviors (e.g., bullying) [10].

School climate and school identification are two distinct but closely related constructs. While school climate focuses on students’ perceptions of the school as a group, school identification reflects the group’s importance to its students [4]. School climate may affect school identification [11]. The Social Identity Theory and Self-Categorization Theory suggest that one would internalize the key features of the group that one values (school climate) into one’s self-identity (school identification) [12]. It is warranted to investigate the joint impacts of school climate as a ‘group concept’ and school identification as a ‘me concept’ on adolescent health. Reliable and valid assessment tools of school climate and school identification are greatly warranted to facilitate such research and foster interventions.

A number of related assessment instruments have been developed, such as the School Climate-Revised [13] and Identification with School Questionnaire [14]. Some researchers have criticized that such instruments are based on different definitions and dimensions, making it hard to reach a consensus [15]. Furthermore, these scales may have mixed-up items of school climate and school identification. This study validates the Chinese version of the 38-item Dual School Climate and School Identification Measure–Student (SCSIM-St38), which was developed in 2017. Its four factors cover school climate (i.e., student–student relationships, staff–student relationships, academic emphasis, and shared values and approach) while the fifth factor assesses school identification [12]. This dual measure of school climate and school identification is advantageous, as it includes an assessment of both the external environment and internal responses. Moreover, the dualism is consistent with the theoretical frameworks of the Ecological Theoretical Model, which explains individual behaviors through the operations of various social subsystems [16], and the Social Identity Theory, which postulates that school climate affects school identification [17]. The tool has been validated among adolescents in Australia [12], Turkey [18], and Chile [19]. The psychometric properties (i.e., reliability, factor structure, and criterion validity) are consistently satisfactory. Such a dual-construct measure has a good potential for facilitating adolescent research, assessing socio-educational school environments, and evaluating related interventions such as those improving school performance and school management and multiple health behavioral changes [20]. A 15-item short-form (i.e., SCASIM-St15) has recently been developed among Chilean secondary school students [20]. The SCASIM-St15 demonstrates the same second-order factor structure and satisfactory reliability and criterion validity; it has the advantage of having fewer items. Notably, both SCSIM-St38 and SCASIM-St15 have not been validated among Chinese adolescents.

Given the background, this study validates the Chinese version of SCASIM-St15 among adolescents in China, examining its psychometric properties including factor structure, measurement invariance, internal consistency, floor and ceiling effects, and external validity. Determinants of multiple health behavioral changes including depressive symptoms, peer victimization, classmate support, and teacher–student relationships were selected as external variables, as the literature has documented their associations with school climate and school identification, both theoretically and empirically [3,5,6,7,9,10,20]. It was hypothesized that a better school climate and stronger school identification are negatively associated with both depressive symptoms and peer victimization and positively associated with both classmate support and teacher–student relationships. Subgroup differences in the subscale scores by sex and school type (including junior middle school students, senior middle school students, and vocational high school students) were also tested.

## 2. Materials and Methods

### 2.1. Participants and Data Collection

The survey was conducted from 20 February to 4 March 2022 in Taizhou city, Zhejiang province, China. Five junior middle schools, three senior middle schools, and one vocational school were conveniently selected and consented to join this study (a total of nine schools). Grade 1 and Grade 2 students from the selected schools were invited to participate in the study. In the classroom setting, the fieldworkers briefed the students about the purpose and logistics of the study. They highlighted the anonymous nature and that return of the completed questionnaire implied informed consent to participate in the study. The participants were reminded that they could quit anytime without being questioned, and there would be no negative consequences for refusals. The students were requested to read such information, which was also printed on the cover page of the questionnaire. They then self-administered the anonymous, structured questionnaire in the absence of teachers. The field workers answered inquiries and cross-checked the completed questionnaires. All students were requested to inform their parents about the study. Parental opt-out was exercised, but no opt-out form was returned from the parents to the teachers. No incentive was given to the participants. This study was approved by the research ethics committee of the corresponding author’s affiliated institution (No. KNLL-20211011002).

A total of 8285 completed questionnaires were collected; 114 (1.4%) and 615 (7.4%) were excluded from data analysis due to having over 20% missing data among all questionnaire items and/or inconsistencies found in some built-in logical checks. The remaining 7556 (91.2%) were valid questionnaires. Regarding this validation exercise, a subsample of 1108 (14.7%) participants was randomly selected from all 7556 valid questionnaires using “Select cases” in SPSS software. This subsample showed a mean (SD; range) age of 15.1 (1.5; 12–19) years; 56.5% were males; and 53.1%, 27.9%, and 19.0% were currently studying at junior middle schools, senior middle schools, and vocational high schools, respectively.

### 2.2. Measures

#### 2.2.1. SCASIM-St15

Similarly to the SCSIM-St38, the SCASIM-St15 comprises five first-order factors, of which four assess school climate (i.e., student–student relations, staff–student relations, academic emphasis, and shared values and approach) and one assesses school identification [20]. The English items (see Table 1) were translated into Chinese and back-translated by two bilingual researchers; the wording of the Chinese version (see Supplementary Materials Table S1) was then finalized by two senior researchers. The items were rated on a 5-point Likert scale (1 = strongly disagree to 5 = strongly agree); higher scores indicated a better school climate or stronger school identification.

#### 2.2.2. External Variables

1. Depressive symptoms: They were assessed using the 9-item Patient Health Questionnaire (PHQ-9), a multipurpose instrument for screening, diagnosing, and monitoring the severity of depression. Its Chinese version has been validated in adolescents and shows good psychometric properties [21,22]. A sample item is “Little interest or pleasure in doing things”. The items were rated with a 4-point Likert scale according to the frequency in the past two weeks (0 = not at all to 3 = nearly every day). The Cronbach’s alpha of the scale was 0.88 in this study.
2. Peer victimization: It was assessed using a 4-item scale [23] that was adapted from the Panel Study of Income Dynamics, Child Development Supplement III. Participants were asked to report the frequencies of four types of peer victimization occurring in the past month, including (1) “picked on you or said mean things to you”, (2) “hit you”, (3) “taken your things, like your money or lunch, without asking”, and (4) “purposely left you out of activities” (0 = never to 4 = always). The English items were translated into Chinese and back-translated by two researchers. The Cronbach’s alpha of this scale was 0.70 in this study.
3. Classmate support: It was assessed using a 3-item scale about perceived emotional support, instrumental support, and positive appraisals from classmates. The items asked about the agreement with these statements: “You would be able to obtain adequate support from your classmates when you need emotional support or talking to someone about your emotions”, “You would be able to obtain adequate support from your classmates when you need practical help (e.g., problem-solving in daily or school life”, and “You would be able to obtain adequate support from your classmates when you need positive appraisals about yourself” (1 = strongly disagree to 7 = strongly agree). Similar items have been used in previous studies [24,25]. The Cronbach’s alpha of this scale was 0.89 in this study.
4. Teacher–student relationship: The item was: “In general, how is your relationship with schoolteachers” (0 = extremely poor to 10 = extremely good).

### 2.3. Statistical Analysis

A confirmatory factor analysis (CFA) with a maximum likelihood with robust standard errors (MLR) estimation was conducted to confirm the second-order factor structure of the SCASIM-St15. Recommended goodness-of-fit statistics and cut-off criteria were: Chi-square/*df* ratio <5.00, both Comparative Fit Index (CFI) and Tucker–Lewis Index ≥0.90, and Root Mean Square Error of Approximation (RMSEA) ≤0.08 [26]. Measurement (configural, metric, and scalar) invariance was further tested using a multigroup CFA to explore whether the factor structure would differ across sex and school types. Measurement invariance would be supported if the difference in CFI (ΔCFI) between the restricted model and baseline model was ≤0.01 [27]. To simplify the modeling process [12,28], the senior middle school group was combined with the vocational high school group to form the ‘senior/vocational high school’ group, which was compared to the junior middle school group. The presence of a floor effect and ceiling effect in the SCASIM-St15 subscales would occur if more than 15% of the participants possessed a minimum or maximum subscale score, respectively [29]. Internal consistency was assessed using Cronbach’s alpha coefficients. External validity was established by inspecting Pearson correlation coefficients between the subscale scores of the SCASIM-St15 and the four external variables (i.e., depressive symptoms, peer victimization, classmate support, and teacher–student relationship). T-test and ANOVA were conducted to test the subgroup differences in the subscale scores of the SCASIM-St15; Cohen’s *d* and Eta Squared were used to demonstrate the effect sizes. CFA was conducted using Mplus 7.0 (Muthén & Muthén, Los Angela, CA, USA), while the other tests were analyzed using SPSS 23.0 (IBM Corp., Armonk, NY, USA). Statistical significance was defined as *p* < 0.05 (two-tailed tests).

## 3. Results

### 3.1. Descriptive Statistics

The mean (SD; range) of all 15 individual items of the SCASIM-St15 and the external variables are presented in Table 1.

### 3.2. CFA of the Second-Order Structure of the SCASIM-St15

**Figure 1:**
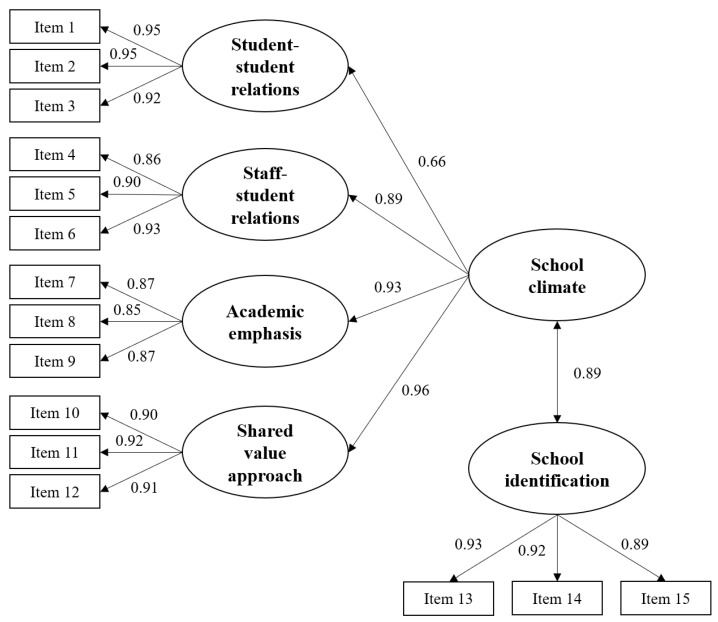
Confirmatory factor analysis of the second-order factor structure of the SCASIM-St15 (Note. Standardized coefficients are reported; all factor loadings are statistically significant with *p*-value < 0.001).

The results are presented in Figure 1. The second-order factor structure yields a satisfactory goodness-of-fit (Chi-square/*df* = 325.33/85 = 3.83; CFI = 0.97; TLI = 0.96; RMSEA = 0.05), with factor loadings ranging from 0.66 to 0.96 (all *p* < 0.001). This factor structure is, hence, acceptable. The correlation matrix of the identified factors is presented in the Supplementary Materials Table S2.

### 3.3. Measurement Invariance

The multigroup CFA on sex invariance shows that: (1) invariance of the factor structure (configural) is supported, as the model fit index for the baseline model is satisfactory (Chi-square/*df* = 55.51/26 = 2.14; CFI = 0.99; TLI = 0.99; RMSEA = 0.05); (2) invariance of the factor loadings (metric) is also supported, as the change in statistics between Model 1 (removing the restrictions on factor loadings) and the baseline model meets the criterion (ΔCFI = 0.99−0.98 ≤ 0.01);(3) the invariance features of the factor loadings and intercepts (scaler) are supported, as the change in statistics between Model 2 (removing the restrictions on both factor loadings and intercepts) and Model 1 also meets the criterion (ΔCFI = 0.98−0.98 ≤ 0.01).

Similarly, the multigroup CFA results show configural invariance between school types (model fit index for baseline model: Chi-square/*df* = 119.52/26 = 4.60; CFI = 0.99; TLI = 0.99; RMSEA = 0.08). Metric and scalar invariance across the school types are also supported as indicated by the ΔCFI ≤ 0.01 in both cases.

### 3.4. Internal Consistency and Floor and Ceiling Effects

The results are presented in Table 2. The Cronbach’s alpha values of the five factors of the SCASIM-St15 range from 0.90 to 0.94. No floor effect was noticed, as the proportions of those reporting minimum scores on the five subscales range from 1.0% to 3.7%. However, obvious ceiling effects were observed; the proportions of those reporting maximum scores on the five subscales range from 27.5% to 38.4%.

### 3.5. External Correlations

The four subscales of school climate (student–student relations, staff–student relations, academic emphasis, and shared values and approach) and the subscale of school identification are all negatively correlated with both depressive symptoms (r = −0.41 to −0.30; all *p* < 0.001) and peer victimization (r = −0.31 to −0.26; all *p* < 0.001). They are positively correlated with both classmate support (r = 0.29 to 0.38; all *p* < 0.001) and teacher–student relationships (r = 0.22 to 0.41; all *p* < 0.001) (see Table 2).

### 3.6. Subgroup Differences by Gender and Type of School

The mean (SD; range) scores of the five subscales of the SCASIM-St15 are presented in Table 3. There are no sex differences in the five subscale scores (all Cohen’s *d* = 0.01; *p* values range from 0.541 to 0.980). The ANOVA results show that the three school types differ statistically in the subscale scores of staff–student relations, academic emphasis, shared values and approach, and school identification but not student–student relations. Specifically, pairwise comparisons (Bonferroni adjustment with *p* < 0.017 indicating statistically significant between-group differences) were conducted, showing that junior middle school students tended to have higher scores in general. Specifically, (1) junior middle school students show higher levels of staff–student relations and school identification than senior middle school students (both *p* < 0.001); (2) all the between-group differences in academic emphasis and shared values and approach are statistically significant, with subscale scores of junior middle school > vocational high school > senior middle school (all *p* < 0.017).

## 4. Discussion

This study validates the SCASIM-St15 among Chinese adolescents studying in junior middle schools, senior middle schools, and vocational high schools. In general, the results demonstrate satisfactory psychometric properties. A stable second-order factor structure including the four domains of school climate (student–student relations, staff–student relations, academic emphasis, and shared values and approach) and school identification was found. Measurement invariance (configural, metric, and scalar) was established across sex and school types. There was adequate internal consistency, an absence of floor effects, and satisfactory criterion validity based on the significant correlations between the subscale scores and the four external variables. The evaluated psychometric properties and related criteria used in this study are consistent with those commonly used in previous scale validation studies [29,30,31,32]. The satisfactory psychometric properties suggest that the tool can be used in various general student populations. This study has hence added to the literature on SCASIM-St15. The tool was developed and mainly used in Western countries [12,17,18,20]; the present validation allows it to be used in Chinese populations.

Notably, all five subscales of the SCASIM-St15 show substantial ceiling effects of over 27%. The observed scores are, in general, higher than those of the Chilean sample using SCASIM-St15 [20]. Some characteristics of Chinese schools might contribute to such high levels of school climate and school identification, including large class sizes (e.g., over 50 students per class), a stronger commitment to education [33,34], respect for teachers and authorities [33,34], more opportunities for bonding between staffs/students and students to promote social harmony [35], and whole-class teaching instead of small-group or individualized teaching [36,37].

This study found that the four school climate subscales and the school identification subscale are negatively correlated with depression and peer victimization and positively correlated with classmate support and teacher–student relationships. Such findings support our hypotheses and corroborate previous studies [3,5,6,7,9,10,20]. The results highlight the potential impact of the school environment on student behavioral health (e.g., school bullying) and mental health (e.g., depression). School climate/identification may also promote factors protecting against depression and school bullying, such as classmate support and teacher–student relationships [38,39,40,41]. Furthermore, school identification shows a stronger effect size on depressive symptoms than social climate, and the reverse occurs for peer victimization. In this case, the ‘me’ concept seems to be more important than the ‘group (school)’ concept, but the preliminary analysis needs further proof. Such findings highlight the usefulness of the SCASIM-St15 in assessing both school climate and school identification. Future studies are warranted to investigate the joint and independent effects of these two constructs using the validated SCASIM-St15.

A comparative analysis shows that the levels of school climate and school identification do not differ significantly between male and female students, which corroborates previous studies conducted on U.S. students [42,43]. Future studies are warranted to confirm such non-significant sex differences, as some researchers argue that girls are more likely than boys to report negative school climate as they might experience institutional gender biases and even sexual harassment from their male peers; such experiences might create unfavorable perceptions toward the school [44]. This study also shows that the levels of school climate and school identification are, in general, higher among junior middle school students than senior middle school students, indicating potential age differences in perceptions. These two groups of students may experience different daily school routines and teacher–student relationships that might affect school climate and school identification [45]. Adolescents may also make finer distinctions regarding specific changes in their environments than children [46]. Such cognitive developments may affect the perceived school climate [47]. Furthermore, it is interesting that the vocational high school students reported higher subscale scores regarding academic emphasis and shared values and approach than mainstream senior middle school students. It is plausible that vocational schools in China tend to present clearer values and approaches related to job preparation.

It is a strength of the study that it includes a wide age span and multiple school types, which increases the scale’s applicability. In particular, very few validations of the SCASIM-St15 have targeted vocational school students. This study, however, has some limitations. First, the Chinese version of the original 38-item SCSIM-St has not been validated in this or any other studies. Second, as the questionnaire was self-administered, reporting bias may exist, as an endorsement of a positive school climate and school identification tends to be socially desirable. Third, the sample was recruited from a convenience sample in a single city in China, while geographical differences occur. In addition, this study was conducted during the pandemic period (February to March 2022). The pandemic might have affected school climate but more on the levels of the subscale scores than its psychometric properties if such is true. Moreover, during the study period, zero COVID-19 cases were then found in Taizhou. Life in Taizhou and the majority of Chinese cities was, hence, very normal, as there were no school closures and no lockdowns. Thus, the pandemic was not a concern regarding the validation exercise. It is actually interesting to apply the scale to places where school suspensions and online teaching were practiced with an aim to understand the impact of the pandemic in future studies.

## 5. Conclusions

To conclude, the Chinese version of the SCASIM-St15 shows acceptable psychometric properties among students of three types of schools in China. Its strength in simultaneously assessing school climate and school identification has been demonstrated. The tool has a good potential to improve social climate and social identification research in China, including both observational studies and intervention studies. More validation in other countries is warranted to confirm or compare the scale’s factor structure.

## Figures and Tables

**Table 1:** Descriptive statistics (n = 1108).

Variables	Range	Mean	SD
**Items of the SCASIM-St15**			
***School climate***			
Student–student relations			
Item 1: Students treat each other with respect	1–5	4.00	1.05
Item 2: Students are fair to each other	1–5	3.97	1.08
Item 3: Students show understanding to each other	1–5	3.94	1.07
Student–staff relations			
Item 4: Staff go out of their way to help students	1–5	4.11	0.95
Item 5: Staff are fair in their dealing with students	1–5	3.88	1.12
Item 6: Staff show understanding to students	1–5	3.90	1.07
Academic emphasis			
Item 7: Teachers challenge students to do better	1–5	3.92	1.01
Item 8: Teachers want every student to do their best	1–5	4.06	0.99
Item 9: Teachers believe that every student can be a success	1–5	3.86	1.07
Shared values and approach			
Item 10: There is a sense that we are all on the same team	1–5	3.74	1.15
Item 11: There is school spirit and pride	1–5	3.80	1.10
Item 12: The school values and goals are well understood	1–5	3.76	1.09
***School identification***			
Item 13: I am happy to be a part of this school	1–5	3.72	1.14
Item 14: I feel a strong connection with this school	1–5	3.59	1.16
Item 15: I identify with this school	1–5	3.83	1.11
**External variables**			
Depressive symptoms	0–27	5.51	5.11
Peer victimization	0–16	1.52	2.09
Classmate support	3–21	13.67	3.93
Teacher–student relationships	0–10	6.25	1.82

Abbreviations: SD—Standard deviation; SCASIM-St15—The abbreviated version of the Dual School Climate and School Identification Measure-Student.

**Table 2:** Internal consistency, floor and ceiling effects, and external correlations.

Variables	Cronbach’s α	Floor Effect (%)	Ceiling Effect (%)	External Correlations			
				Depressive Symptoms	Peer Victimization	Classmate Support	Teacher–Student Relationships
**School climate**							
Student–student relations	0.96	1.7	38.4	−0.33 ***	−0.31 ***	0.38 ***	0.22 ***
Staff–student relations	0.92	1.0	33.3	−0.32 ***	−0.28 ***	0.29 ***	0.41 ***
Academic emphasis	0.90	1.3	29.8	−0.30 ***	−0.27 ***	0.31 ***	0.40 ***
Shared values and approach	0.94	2.5	29.1	−0.37 ***	−0.29 ***	0.33 ***	0.35 ***
**School identification**	0.94	3.7	27.5	−0.41 ***	−0.26 ***	0.36 ***	0.38 ***

*** *p* < 0.001.

**Table 3:** Subgroup analysis by sex and school types.

Variables	Range	Overall	Sex			School Type			
			Male	Female	*p* (Cohen’s *d*)	Junior Middle School	Senior Middle School	Vocational High School	*p* (Eta Squared)
		Mean, SD	Mean, SD	Mean, SD		Mean, SD	Mean, SD	Mean, SD	
**School climate**									
Student–student relations	3–15	11.92, 3.07	11.91, 3.14	11.90, 2.99	0.980 (0.01)	12.09, 3.18	11.67, 2.80	11.81, 3.09	0.119 (0.004)
Staff–student relations	3–15	11.88, 2.92	11.92, 2.98	11.81, 2.85	0.541 (0.01)	12.27, 2.87	11.24, 2.81	11.76, 3.01	<0.001 (0.023)
Academic emphasis	3–15	11.83, 2.79	11.85, 2.90	11.80, 2.66	0.769 (0.01)	12.31, 2.66	11.19, 2.67	11.43, 3.10	<0.001 (0.035)
Shared values and approach	3–15	11.30, 3.14	11.29, 3.30	11.29, 3.95	0.970 (0.01)	11.88, 3.07	10.31, 2.98	11.14, 3.22	<0.001 (0.046)
**School identification**	3–15	11.13, 3.23	11.10, 3.38	11.14, 3.04	0.811 (0.01)	11.64, 3.26	10.25, 3.04	11.00, 3.13	<0.001 (0.034)

Note. Cohen’s *d* values of 0.20, 0.50, and 0.80 indicate small, medium, and large effect size, respectively. Eta squared values of 0.010, 0.059, and 0.138 indicate small, medium, and large effect size, respectively.

## Data Availability

Data is available upon reasonable request.

## Supplementary Materials

The following supporting information can be downloaded at: https://www.mdpi.com/article/10.3390/ijerph192416535/s1, Table S1: The Chinese version of the Abbreviated Version of the Dual School Climate and School Identification Measure-Student (SCASIM-St15); Table S2: Correlation matrix among the factors of SCASIM-St15.
