# Supplementary material for: Psychometric Properties of the Abbreviated Version of the Dual School Climate and School Identification Measure–Student (SCASIM-St15) among Adolescents in China
Source: Int J Environ Res Public Health. 2022 Dec 9;19(24):16535. doi: 10.3390/ijerph192416535 (PMC9779404; doi:10.3390/ijerph192416535)
Supplement: Supplementary file 1 [file ijerph-19-16535-s001.zip › ijerph-1950836-supplementary.pdf]

Supplementary material

Table S1. The Chinese version of the Abbreviated Version of the Dual School Climate and School Identification Measure-Student (SCASIM-St15)

Subscale	English items	Chinese items
Subscale 1: Student-student relations	Item 1. Students treat each other with respect	同学之间是互相尊重的
	Item 2. Students are fair to each other	同学之间是公平地对待对方的
	Item 3. Students show understanding to each other	同学之间是互相理解的
Subscale 2: Staff-student relations	Item 4. Staff go out of their way to help students	老师是尽力帮助学生的
	Item 5. Staff are fair in their dealing with students	老师是公平地对待学生的
	Item 6. Staff show understanding to students	老师会表达对学生的理解
Subscale 3: Academic emphasis	Item 7. Teachers challenge students to do better	老师会给学生挑战，使他们做得更好
	Item 8. Teachers want every student to do their best	老师想每一个学生都做到最好
	Item 9. Teachers believe that every student can be a success	老师相信每一个学生都会成功
Subscale 4: Shared values and approach	Item 10. There is a sense that we are all on the same team	学校有大家都属于同一个团队的感觉
	Item 11. There is school spirit and pride	学校是具有学校精神和自豪感的
	Item 12. The school values and goals are well understood	学校有大家都清楚理解的价值观和目标
Subscale 5: School identification	Item 13. I am happy to be a part of this school	我很开心自己是学校的一份子
	Item 14. I feel a strong connection with this school	我觉得自己与学校紧密相连
	Item 15. I identify with this school	我认同自己是学校的一份子

Table S2 Correlation matrix among the factors of SCASIM-St15

	1	2	3	4	5
**School climate**					
1. Student-student relations	-				
2. Staff-student relations	0.56***	-			
3. Academic emphasis	0.55***	0.85***	-		
4. Shared values and approach	0.61***	0.76***	0.80***	-	
**5. School identification**	0.57***	0.69***	0.72***	0.85***	-

Note. ***, $p<0.001$.
